# Endogenous endophthalmitis caused by *Pseudomonas aeruginosa *in a preterm infant: a case report

**DOI:** 10.1186/1757-1626-2-9304

**Published:** 2009-12-10

**Authors:** Sofia Figueiredo, Anabela João, Manuela Mateus, Rosário Varandas, Leonor Ferraz

**Affiliations:** 1 Department of Pediatrics - Neonatal Unit, Centro Hospitalar Vila Nova de Gaia/Espinho, EPE, Rua Conceição Fernandes, 4434-502 Vila Nova de Gaia, Portugal; 2 Department of Ofthalmology, Centro Hospitalar Vila Nova de Gaia/Espinho, EPE, Rua Conceição Fernandes, 4434-502 Vila Nova de Gaia, Portugal

## Abstract

Endophthalmitis is an infection of the vitreous or aqueous humor of the eye. Although it rarely occurs in the neonatal period it has been previously diagnosed in preterm infants.

Endogenous endophthalmitis is when eye infection is secondary to septicemia and represent 20% of the cases of endophthalmitis. *Pseudomonas aeruginosa *is responsible for more than 75% of invasive neonatal eye infections. The course of pseudomonal endophthalmitis is typically fulminant, developing over hours even in early diagnosis. For survivors, the usual result is blindness of the affected eye.

We report the case of a preterm infant who developed septicemia and was later diagnosed as having a pseudomonas endophthalmitis.

## Introduction

Endophthalmitis is results from a bacterial or fungal infection of the vitreous or aqueous humor of the eye. It is rare in the neonatal period only occurring in susceptible individuals such as preterm infants [1,2].

Endogenous endophthalmitis represents 20% of the cases of endophthalmitis and occurs when eye infection is secondary to septicemia [3]. *Pseudomonas aeruginosa *is an aggressive gram-negative bacillus and is responsible for more than 75% of invasive neonatal eye infections [1,2,4].

Acute bacterial endophthalmitis is a vision-threatening condition and must be managed as an emergency.

We report the case of a preterm infant who developed septicemia and was later diagnosed as having a pseudomonas endophthalmitis

## Case presentation

A female infant was born at 32 weeks' gestational age by caesarean delivery. The caesarean was performed after detection of signs of fetal distress by cardiotocography. The mother was initially admitted with premature labor and subsequently treated with two doses of antenatal steroids. The baby weighted 1660 g and had Apgar scores of 7, 7 and 7 at 1, 5 and 10 minutes, respectively.

On admission the newborn presented moderate respiratory distress syndrome. No other relevant physical signs were observed. Chest radiography revealed diffused reticulogranular pattern of the lung. She started on Infant Flow Driver nasal continuous positive airway pressure (nCPAP) and received one dose of surfactant. An evaluation for sepsis was performed. In view of the history of premature labor, she was treated with intravenous (i.v.) ampicillin and gentamicin and completed a nine day course despite negative blood cultures.

The newborn was maintained on nCPAP until day 3 (FiO2 max: 32%). Afterwards, she required O2 supplementation (0,5-1 L/min) until the fifth day of hospital stay.

On the third day a 3/6 systolic murmur was noted after which an echocardiogram revealed patent ductus arteriosus (PDA) and patent foramen ovale (PFO). She received a 3 days treatment with i.v. administration of a prostaglandin inhibitor (indomethacin). Closed ductus diagnosis was further confirmed by control echocardiography.

Due to the fact that the newborn developed physiological jaundice phototherapy treatment was performed from day 3 to day 5.

Oral feeds were introduced on day 3 and established by day 20. From day 2 to day 21 she was on parenteral nutrition through a peripheral catheter.

On day 9 of admission the baby's condition deteriorated acutely: she was lethargic, with a *subicteric *tint appearance and presented important gastric aspirates. A full infection screen followed by an initial treatment with i.v. amicacin and vancomycin were performed. Hematologic data revealed anemia and thrombocytopenia (Hemoglobin - 105 g/L; Leucocytes counts - 15.9*10^9^/L with 60% neutrophilis and 36% lynfocytes, platelet count - 102*10^9^/L). The value of C-reactive protein was 174 mg/L. The analysis of cerebrospinal fluid was normal. On the same day her mother reported a post-cesarean wound infection and a wound swab was taken.

On day 10 of admission conjunctival erythema associated with purulent discharge was observed on the left eye of the newborn. At this stage it was not possible to visualize the iris and the pupil due to the development of corneal clouding. Conjunctival swabs were sent for microbiological investigations and the result was negative for the presence of microorganisms. After an urgent ophthalmological consultation an endophthalmitis with hypopyon was diagnosed. The patient underwent hourly topical treatment with tobramicine. Ocular echography revealed alterations that were suggestive of vitreitis and retinal detachment.

Blood culture revealed *Pseudomonas aeruginosa*. Antibiotherapy was changed to amicacin and ceftazidime and carried out for 21 days. Cerebrospinal fluid culture was negative. The sample from mother's caesarean wound also grew *Pseudomonas aeruginosa*.

The baby showed progressive clinical improvements. However the intensive systemic and topical antibiotic therapy did not prevent intraocular infection deterioration. Indeed proptosis and spontaneous corneal perforation with a purulent discharge occurred. There was no visual potential. On day 25 an evisceration was performed and a silicon ocular prosthesis was set. Histological examination of the removed eye showed a supurative inflammatory infiltrate of choroid and retina. This is consistent with an endophthalmitis's lesion.

Observation of the right eye did not show signs of development of Retinopathy of Prematurity.

## Discussion

Endophthalmitis can be classified as either endogenous or exogenous, depending on the route of infection. Exogenous endophthalmitis contributes to 80% of the cases of endophthalmitis and occurs when the eye infection develops as a result of corneal infection, perforating injury or intraocular surgery [3-5]. Endogenous endophthalmitis results from hematogenous spread to the eye secondary to septicemia [4]. We report a case of a newborn that showed signs of sepsis before ocular's manifestations of endophthalmitis and presented an intact corneal surface at an initial stage. These observations were consistent with a diagnostic of endogenous endophthalmitis [3,5]. Conjunctival swabs were negative for the presence of pseudomonas whereas blood cultures were positive. These results also suggested an endogenous source of infection. In unilateral cases of endogenous endophthalmitis, the right eye is twice as likely to become infected as the left eye, probably because of its greater proximity to direct arterial blood flow [6,7]. In our case the affected eye was the left one.

Organisms previously reported as pathogenic agents of endophtalmitis include *Pseudomonas aeruginosa*, group B *streptococci*, *Haemophilus influenzae *type b, *Staphylococcus aureus*, *Salmonella enteritis*, *Streptococcus pneumoniae*, *Neisseria meningitis *and *Candida *species [3]. Pseudomonas is a virulent organism that produces proteoglycanolytic enzymes that are able to break down tissue barriers as the corneal stroma and conjunctival blood vessels [1,3]. Although our patient started at an early stage intensive intravenous topic antibiotherapy, deterioration of the intraocular infection was observed. This could be justified by the high virulence of Pseudomonas.

Premature infants are particularly vulnerable to *Pseudomonas *infections [1]. They are immunocompromised and they often have multiple systemic problems related to prematurity [3]. Moreover they are depended on a range of different equipments (humidifiers, incubators, respirators and suction apparatus) that are required to keep them alive but that can also be a source of nosocomial infection (when contaminated) [1,3]. In our case the source for the eye infection could have been the mother's post-caesarean wound infection caused by *Pseudomonas aeruginosa*. Furthermore, neonates are unable to complain of eye pain or of decreasing vision, making early diagnosis more difficult [3,5,8]. An eye examination is vital in the septic neonate and should be included by neonatologists as part of the systemic work-up [2].

Physical findings are correlated with the structures involved and with the degree of infection. These include: eyelid swelling and erythema, corneal edema and infection, injected conjunctiva and sclera, purulent discharge, hypopyon (layering of inflammatory cells and exudate in the anterior chamber), vitreitis, vitreal mass and debris, reduced or absent red reflex, limited view of the fundus, proptosis (a late finding in panophthalmitis) [7,9]. Additionally an ultrasonography could be necessary to establish the diagnosis.

Although organisms can be occasionally cultured from aspirated vitreous fluid, the diagnosis is established most frequently from blood cultures [7,9]. In our case aspiration of vitreous fluid was not performed. Culture of the purulent discharge can be useful but is less reliable for the identification of the causative organism [4]. Nevertheless, growth of *P. aeruginosa *on discharge from an eye of a sick child should alert the clinician to the risk of life threatening infections [4].

The most appropriate treatment for endogenous endophthalmitis is a combination of intravenous vancomycin and third-generation cephalosporin or aminoglycoside. As intraocular accumulation of intravenous antibiotics is poor, the use of intravitreal antibiotics is indicated [4]. However some case series had shown a poor visual outcome despite treatment with intravitreal antibiotics [7,10]. In our case the ocular echography that was performed when diagnosis was made revealed retinal detachment, which contraindicate the use of intravitreal antibiotics. Topical treatment may be also used, but not as sole treatment [2,4]. Evisceration may be necessary in life-threatening sepsis or in cases of endphthalmitis unresponsive to antibiotics (as happened with our newborn) [1].

The course of pseudomonal endophthalmitis is typically fulminant developing over hours. Morbidity and mortality in pseudomonal endophthalmitis are high, even in early diagnosis [4]. For survivors, the usual result is blindness of the affected eye [2,4].

## Consent

Written informed consent was obtained from the patient's parents for publication of this case report. A copy of the written consent is available for review by the Editor-in-Chief of this journal.

## Competing interests

The authors declare that they have no competing interests.

## Authors' contributions

SF did the literature search and wrote the manuscript. AJ has been involved in critical revision of manuscript. RV made the diagnostic and ophthalmologic follow-up, and performed the evisceration. MM and LF had been involved in drafting of the manuscript. LF obtained written consent. All authors read and approved the final manuscript.
